# Supplementary material for: Children and young people’s contributions to public involvement and engagement activities in health-related research: A scoping review
Source: PLoS One. 2021 Jun 9;16(6):e0252774. doi: 10.1371/journal.pone.0252774 (PMC8189547; doi:10.1371/journal.pone.0252774)
Supplement: S1 Appendix — (PDF) [file pone.0252774.s001.pdf]

S1 Appendix

MEDLINE (via EBSCOhost) Search Strategy

1 AB (“Patient and Public Involvement" OR "Patient and Public Involvement and Engagement" OR “Patient Engagement” OR “Patient Participation” OR “Community Participation”) OR TI ("Patient and Public Involvement" OR "Patient and Public Involvement and Engagement" OR “Patient Engagement” OR “Patient Participation” OR “Community Participation”) (7280)

2 AB (Patient* OR public) N3 (participat* OR involve* OR consult* OR collaborat* OR engage* OR co-produc* OR "Service User*" OR "advisory group" OR "advisory panel" OR "advisory board*" OR "advisory committee*" OR "User involvement" OR "user led" OR "user-led" OR stakeholder OR partners* OR client OR ambassador) OR TI (Patient* OR public) N3 (participat* OR involve* OR consult* OR collaborat* OR engage* OR co-produc* OR "Service User*" OR "advisory group" OR "advisory panel" OR "advisory board*" OR "advisory committee*" OR "User involvement" OR "user led" OR "user-led" OR stakeholder OR partners* OR client OR ambassador) (130,875)

3 1 OR 2 133,405

4 AB (paediatric* OR pediatric* OR child* OR adolesce* OR teen* OR youth OR "young people" OR "young person" OR "young adult*") OR TI (paediatric* OR pediatric* OR child* OR adolesce* OR teen* OR youth OR "young people" OR "young person" OR "young adult*") 1750425

5 AB (research OR study OR project* OR studies OR “Research Design” OR “Health Services Research” OR “Health Research” OR “Research Impact” OR Co-design OR Co-research) OR TI (research OR study OR project* OR studies OR “Research Design” OR “Health Services Research” OR “Health Research” OR “Research Impact” OR Co-design OR Co-research) (1003435)

6 3 AND 4 AND 5 (7212)

7 Limit to 2000 -2019, academic journals, and English language (5828).
