# Supplementary material for: Children and young people’s contributions to public involvement and engagement activities in health-related research: A scoping review
Source: PLoS One. 2021 Jun 9;16(6):e0252774. doi: 10.1371/journal.pone.0252774 (PMC8189547; doi:10.1371/journal.pone.0252774)
Supplement: S1 Table — (PDF) [file pone.0252774.s002.pdf]

S1 Table: Data Extraction reporting Public and Patient Involvement and Engagement (PPIE) for studies included in the scoping review

Author, Year, Country	Aim of PPI	PPI Term, Definition & Underpinning Concepts/Theory	PPI Population	Design and stages of involvement	Methods by which PPI was evaluated	Evidence of impact of PPI	Conclusions & lessons learned from PPI	Limitations related to PPI	Recommendations arising from PPI
Beatriz et al. (2018), USA	**Aim:** To go beyond individual prevention tactics to build a community-wide, rigorously researched teen dating violence prevention model.	**Term used:** Peer researchers. **Definition:** NR **Concepts/Theory:** Youth Participatory Action Research.	**PPI (YP):** Peer Researchers (n=9; 18-23yrs, Black or Latino). **PPI (Others):** N/A[1].	Training, developing materials, collecting and analysing data.	Internal. Reflection on contributions of peer researchers.	**Impact on Research:** The inclusion of youth Peer Researchers is both acceptable and feasible. Framework strengthens teams' ability to design and conduct a thoughtful and rigorous study. **Impact on Young People:** Helped to develop their capacity and skills for future research, and professional efforts. **Impact on Policy:** NR.	**Conclusions:** Young adult peer researchers shaped study instruments and were often more familiar with intervention settings than researchers. Their suggestions were frequently very useful to both the research and program teams. **Lessons learned:** Need for support and flexibility, communication and flexibility, contextualising research challenges for youth researchers.	NR[2]	The used of the framework is useful in an interdisciplinary evaluation team.
Best et al. (2017), N. Ireland.	**Aim:** To test the feasibility and process of Participatory Theme Elicitation (PTE).	**Term:** Patient and Public Involvement (PPI). **Definition:** NR. But set within wider context of co-production. **Concepts/Theory:** Participatory Theme Elicitation.	**PPI (YP):** Young advisors (n=8) representing those physically active and inactive (12–14yrs; 4 male, 4 female). **PPI (Others):** N/A.	Analysis of data.	NR	**Impact on Research:** Proof of concept demonstrated. Recruitment, retention and training of YAP members appeared successful. **Impact on Young People:** NR. **Impact on Policy:** NR.	**Conclusions:** Proof of concept demonstrated through the ability of thematic patterns to be generated from PTE groupings. Recruitment, retention and training of YAP members appeared successful. **Lessons learned:** A strength of PTE is that its implementation requires little prior knowledge or specialist skills on behalf of users or researchers.	Possibility of selection bias in selection of excerpts shared. Difficult to claim data saturation using only 40 excerpts.	Future PTE research should consider greater involvement of users, what is an 'appropriate' and 'sufficient' number of excerpts, the number of sorters, the number of excerpts, the training time needed for co-researchers and the use of alternative algorithms to produce groupings.
Boote et al. (2016), UK.	**Aim:** To involve children with asthma in the design of a trial intervention to reduce unscheduled medical contacts in children with asthma.	**Term:** PPI. **Definition:** PPI classified into consultation, collaboration or user-led (Fleming and Hudson, 2009). **Concepts/Theory:** NR.	**PPI (YP):** Event 1: Children (n=5) with asthma (7-14yrs; 4 girls). Event 2: Children (n=4) with asthma (11yrs and under; 2 siblings; 3 girls; 2 Caucasian, 2 Asian). **PPI (Others):** N/A.	Research design, development of documents and design of logo	NR	**Impact on Research:** Reorientation to the study, ahead of the discussion that followed. **Impact on Young People:** NR. **Impact on Policy:** NR.	**Conclusions:** The work contributes to the small but growing number of examples of PPI in trial design and conduct. **Lessons learned:** Inconsistency of attendance between events was not problematic and allowed introduced of fresh perspectives.	Number of people in the consultations small due to difficulties recruiting people to PPI. Children in first event may have known facilitators.	Researchers should consider holding post-award, pre-commencement PPI consultation events, as they provide a useful means of providing feedback to lay people on how their input has contributed.
Brady et al. (2018), UK.	**Aim:** To explore whether and how young drug and alcohol service users could be meaningfully and effectively be involved in an RCT.	**Term:** Involvement. **Definition:** Discussed in terms of "research [...] carried out 'with' or 'by' members of the public rather than 'to', 'about' or 'for' them"	**PPI (YP):** Young advisors (n=17, 16-21yrs; 12 female, 5 male). **PPI (Others):** N/A.	Involvement in all three phases of study as advisors.	Internal feedback.	**Impact on Research:** Opportunity to reflect on how involvement was operationalised and to reflect on the learning that emerged. **Impact on Young People:** Young advisors spoke about the benefits of involvement for	**Conclusions:** Involvement of young people needs to be dynamic, flexible and sensitive. Engagement with services was crucial in recruiting young people and supporting their engagement. The dominant discourses and cultures of	NR	The flexible and young people-centred model for involvement which emerged from this work provides a template for a different approach.

[1] N/A - Not applicable
[2] NR - Not reported

		(INVOLVE, 2016).<br>**Concepts/Theory:** NR.				their wider peer group as well as personal benefits, including being able to use difficult personal experiences to create positive changes.<br>**Impact on Policy:** NR.	health services and research often do not sit easily with partnership initiatives with young people.<br>**Lessons learned:** Time taken to establish a group of young advisors meant it was difficult to involve young people in the initial stage. Researchers to be flexible in response to young people's personal circumstances. Danger of young people in drug and alcohol research being unintentionally disaffected from involvement.		
Byrne (2019), Ireland	**Aim:** To increase the likelihood of developing a feasible, implementable, applicable, and effective intervention.	**Term:** Stakeholder engagement (SE).<br>**Definition:** An umbrella term that encompasses activities, including patient and public involvement (PPI) and patient engagement.<br>**Concepts/Theory:** NR.	**PPI (YP):** 8 members of a young adult panel (18-25yrs) with T1D.<br>**PPI (Others):** N/A.	Developing materials and dissemination.	NR.	**Impact on Research:** Ensured that surveys acceptable to younger adults and dissemination methods increased chances of the surveys reaching young adults.<br>**Impact on Young People:** NR.<br>**Impact on Policy:** NR.	**Conclusions:** Integrating engagement approach was critical to delivering the final product.<br>**Lessons learned:** Engage young people early in the research process and regularly update them on progress. It takes time to build trust and relationships. Engagement requires commitment and flexibility from the research team. Stakeholders should have protected time within research meetings. Abbreviations and technical language should be avoided. Training should be provided to enable participation. Delivering research impact can be a slow process. A neutral local "knowledge broker" can be valuable.	NR.	Future research is needed to develop a solid evidence base for the impact of SE and to estimate where engagement and involvement have the greatest impact.
Carroll et al. (2018), New Zealand.	**Aim:** To foreground the voices of disabled young people.	**Term:** Advisory group.<br>**Definition:** NR.<br>**Concepts/Theory:** NR.	**PPI (YP):** Advisory group of young disabled people. No information provided on number or age range.<br>**PPI (Others):** N/A.	Designing data collection tools	NR.	**Impact on Research:** Change in study age range and data collection tools resulting from feedback.<br>**Impact on Young People:** NR.<br>**Impact on Policy:** NR.	**Conclusions:** Research methods need to be applied flexibly, negotiated and adapted to maximise each young person's participation.<br>**Lessons learned:** A toolbox approach is proving vital to achieving this study's overarching goal.	NR.	A toolbox approach to canvas the diverse voices of mobility, hearing and vision-impaired young people in NZ increases opportunities for their effective community participation.
Chopel et al. (2019), USA.	**Aim:** To explore how e-cigarettes marketed and promoted to youth and how these products are perceived by youth in Oakland?	**Term:** Community-based participatory research (CBPR).<br>**Definition:** CBPR is action-oriented, equitable, and serves as both an intervention and an investigation.	**PPI (YP):** 5 young people (15-24yrs). Two left before study completion to attend college.<br>**PPI (Others):** N/A.	Developing research questions, designing methods, data collection, analysis, and dissemination.	NR (although authors reflect on PPI process in discussion).	**Impact on Research:** Evolution of research aim, influence in terms of methods and introduction of alternative geo-narrative approach.<br>**Impact on Young People:** Training in content, research and communication skills. Developed as advocates in	**Conclusions:** Using a CBPR approach ensured flexibility and responsiveness.<br>**Lessons learned:** Engaging youth as partners in a CBPR study is complex and requires a lot of forethought, planning, and strategy, as well as flexibility and adaptation.	Delays resulted in high turnover in the youth research team. Much of the study resources spent building capacity of youth who were not fully	Future CBPR studies should incorporate safety planning. A two-part training strategy would enable youth to explore various avenues of change-making and embody the

		**Concepts/Theory:** Empowerment theory.				their communities and in local advocacy campaigns. Negative impacts included risks to youth safety. **Impact on Policy:** Two youth became strong asset in the advocacy strategy, and two local ground-breaking ordinances they advocated for passed.		able to engage in the process.	connection between research and action.
Coad (2012), England.	**Aim:** To explore the beliefs about health.	**Term:** Co-researchers. **Definition:** NR. But broad discussion about participation and participatory methods encouraging engagement of young people in research. **Concepts/Theory:** Participatory methodology.	**PPI (YP)**: Young people (n=4, 15-17yrs; 3 females, 1 male). **PPI (Others):** N/A.	Supporting workshops, leading workshop.	NR.	**Impact on Research:** NR. **Impact on Young People:** YP appeared to enjoy being engaged in being co-researchers. **Impact on Policy:** NR.	**Conclusions:** Young people enjoyed being co-researchers and felt comfortable being part of a small peer research group. **Lessons learned:** The initial role was limited but YP pushed for increased participation.	NR.	NR.
Collin & Swist (2016), Australia.	**Aim:** To explore the socio-technical networks and practices of young people.	**Term:** Participation/Participatory design (PD). **Definition:** PD means of expressing, surfacing and supporting engagement with youth perspectives in research and design projects. **Concepts/Theory:** Participatory Design methodology, infrastucturing and attachments.	**PPI (YP)**: Young people (inferred to be 12-18yrs). In Yr 1 (n=>140) involved in participatory research and in Yr 2 (n=163) involved in research & design activities. **PPI (Others):** N/A.	Design, conceptualising, peer research activities, workshops.	NR.	**Impact on Research:** Young people contributed to campaign that is more inclusive of young people's views and experiences. **Impact on Young People:** Connectivity and bonds built with concepts and one another. **Impact on Policy:** NR.	**Conclusions:** While the principles of PD called for inclusivity, recognition of performativity and the negotiation of valued futures, these were in no way guaranteed. Engagement with young people was enabled from disrupting assumptions and strategies based on pre-conceived ideas of authority, expertise and solutions. **Lessons learned:** The embodiment of context, the enactment of creativity and the emergence of connectivity offer novel insights on youth participation in complex research projects.	Logistical challenges in aligning research and co-design activities with young people placed strain on project budgets and timelines. Translation of youth perspectives by various adult researchers produced diverse views on what decisions needed to be made and by whom.	The use of PD could contribute to the promotion of a collaborative 'logic of care' rather than simply an individualised 'logic of choice' in the development of online campaigns.
Cooper et al. (2017), UK.	**Aim:** To collate information to inform the development of the definitive study.	**Term:** PPI. **Definition:** NR. **Concepts/Theory:** NR.	**PPI (YP):** NR **PPI (Others):** N/A.	Designing study and presentation of materials.	NR.	**Impact on Research:** Influenced decisions on co-primary outcome measures of appearance and infection rates, use of 3-point scale, presentation of patient information. **Impact on Young People:** NR. **Impact on Policy:** NR.	**Conclusions:** The full NINJA study objectives were modified, and a follow-up regime and content designed to suit this very specific patient population was developed. Solutions offered by children and parents can be incorporated into trial design at an early stage. **Lessons learned:** NR	NR.	NR.
Costello & Doris (2019), Ireland.	**Aim:** To develop a bespoke PPI initiative to engage and learn directly from children and	**Term:** PPI. **Definition:** Mechanism for mutual and reciprocal learning with the goal	**PPI (YP):** Young people with arthritis (n=9; 10-14yrs, median 14yrs). Siblings	Co-design at every stage.	Internal. Combination of both leading and lagging indicators as	**Impact on Research:** NR. **Impact on Young People:** NR. **Impact on Policy:** NR.	**Conclusions:** The methodological approach was received positively by both researchers and young people. The interactive nature and	The research seminar was not accessible to patients with complex medical	Engaging early, considering the needs of the community and developing appropriate

	adolescents living with arthritis.	of improving their research relevance and enhancing responsible research practices. **Concepts/Theory:** NR.	(n=6,10-17yrs, median 14yrs). Overall, 11 female, 4 male. **PPI (Others):** Parents (n=2).		our measures of effectiveness (MOE). Did not use a formal questionnaire approach instead, using MOE from public postings on social media channels.		learning through doing approach have led to calls from attendees, parents for future interactive, collaborative approaches to opening research to young people. **Lessons learned:** The upskilling for a researcher to feel comfortable to meaningfully engage in PPI are often overlooked. More groundwork may need to be done to help young people feel comfortable to fully engage.	needs. The seminar was advertised only within iCAN networks. Baseline attitudes to research and research involvement not measured prior to attendance.	involvement methodology can enable involvement in pre-clinical research.
Curtin (2007), Australia.	**Aim:** To ensure that research was appropriately designed and that the interests and abilities of the participants were considered.	**Term:** Participation. **Definition:** Children being consulted on matters that affect them and being given adequate information to be able to form an opinion…making choices…influencing decisions, contributing to understanding and solution of social issues. **Concepts/Theory:** NR.	**PPI (YP):** Adolescent siblings. No information provided on number or age range. **PPI (Others):** N/A.	Developing ideas and materials.	NR.	**Impact on Research:** The young people made excellent suggestions for the wording and layout of the questionnaire. **Impact on Young People:** NR. **Impact on Policy:** NR.	**Conclusions:** PPI improved the design of materials and tools. Those who participated in the research projects were consulted but they did not lead or initiate. Projects in which CYP are active participants in all the research stages will lead to more relevant findings, a better understanding of their world, and provide a more authentic voice and identity. **Lessons learned:** In future research we need to work more with CYP in the planning and design stages.	Still a long way to go in facilitating the participation of CYP in research.	An empowerment discourse of childhood needs to be adopted with active listening and acting on what CYP are truly saying.
Dovey-Pearce et al. (2019), UK.	**Aim:** To provide a young people's perspective and to oversee the governance and delivery of the Transition programme.	**Term:** Patient and Public Involvement (PPI). **Definition:** PPI refers to the roles for service users and members of the public in defining, delivering and disseminating research. Includes activities on a continuum from consultative tasks, through to "partnership working" to service-user- led initiatives (INVOLVE). **Concepts/Theory:** NR.	**PPI (YP):** Young people, (n=approx. 20; 15-20yrs) with experience of accessing healthcare services and with experience of living with physical/ developmental conditions. **PPI (Others):** N/A**.**	Overseeing delivery and governance of the research programme, process evaluation.	NR.	**Impact on Research:** NR. **Impact on Young People:** CYP and researchers became a team together. **Impact on Policy:** NR.	**Conclusions:** An overly prescriptive use of guidance to structure involvement work should be guarded against, as it does not show us how to manage all various challenges and emergent opportunities. **Lessons learned:** An agile, reflexive skill set is needed.	NR.	Agile models of working and the potential skills needed, quickly brings people with varied expertise together.
Forsyth et al. (2019), UK.	**Aim:** To obtain the views of CYP on service development plans, the design of generic facility and research participant	**Term:** PPI. **Definition:** Research carried out 'with' or 'by' members of the public rather than 'to', 'about' or 'for' them.	**PPI (YP):** CYP attending Children's Board (n=6, 5-16yrs, median 9yrs). YPAG: No	Informing study design and materials.	NR.	**Impact on Research:** NR. **Impact on Young People:** NR. **Impact on Policy:** NR.	**Conclusions:** This was not a collaborative evaluation; no CYP were co-researchers, therefore potentially important questions could have been missed.	Sample size is extremely small, which limits the findings. No theoretical frameworks were	To have incorporated a more critical review of impact, an evaluation methodology would have had to be

	information, and the suitability of metabolic measurement equipment for CYP use.	(INVOLVE).<br>**Concepts/Theory:** NR.	information on number or age.<br>**PPI (Others):** N/A.				**Lessons learned:** Significant consideration should be given before selecting Advisory Group formats as it is a relatively static approach.	employed. Participants were reviewing past experiences therefore responses are subject to recall bias. CYP were interviewed with their parents, which could have affected their responses.	conceived at the outset. A more robust and meaningful measure would assess the level of co-production.
Funk (2012), Canada	**Aim:** To explore feasibility of youth to co-facilitate the focus groups and what role the youth would be able to take in the study.	**Term:** Participation/Participatory Research.<br>**Definition:** Involvement of youth with personal experience and insights as co-researchers and implementers.<br>**Concepts/Theory:** NR.	**PPI (YP):** Young people (n=6, 19-24yrs; 3 female; 1 identified as having Aboriginal ancestry; 2 had previously injected drugs).<br>**PPI (Others):** N/A.	Co-researchers involved throughout the study.	NR.	**Impact on Research:** Young people's realities and interpretation of the findings is based on experience.<br>**Impact on Young People:** Public speaking experience, research skills, and opportunities to network leading to employment opportunities, appreciation of services available and the gaining of confidence to advocate for themselves and others.<br>**Impact on Policy:** NR.	**Conclusions:** The YIP Project was successful in being a highly participatory research project. In a safe and open environment, youth felt comfortable to question and take on initiatives that went beyond the academic researcher's initial expectations.<br>**Lessons learned:** Flexibility in movement between levels of participation allowed youth to determine their own level of involvement.	It was not feasible to fully involve the youth in all aspects of the YIP project, as many methodological techniques were too advanced and not all youth were interested or capable.	To ensure that youth "truly" participate and determine their own level of participation we recommend that future studies include frequent team-building exercises and evaluations.
Griffiths et al. (2018), UK.	**Aim:** To increase relevance, validity and interpretation, as well as providing research capacity and personal growth.	**Term:** PPI.<br>**Definition:** NR.<br>**Concepts/Theory:** NR.	**PPI (YP):** Main group - Young people with a long-term health condition (n=4, 16-25yrs).<br>PPI events - (n=39, 14-18yrs).<br>**PPI (Others):** N/A.	Project management group and two two-day PPI workshops.	NR (although researcher and young researcher reflections included in report).	**Impact on Research:** Young people influenced research design and interpretation of data.<br>**Impact on Young People:** NR.<br>**Impact on Policy:** NR.	**Conclusions:** NR.<br>**Lessons learned:** NR.	NR.	NR.
Hannon et al. (2018), USA.	**Aim:** To codesign a clinic intervention using shared decision making for addressing diabetes self-care with an adolescent patient and parent advisory board.	**Term:** Co-design.<br>**Definition:** NR.<br>**Concepts/Theory:** Systems design.	**PPI (YP):** Young people with T1 diabetes (n=6; 12-16yrs, median 14.6; 4 male) who were members of patient advisory group.<br>**PPI (Others):** Parents.	Establishing desired intervention outcomes, intervention feasibility and acceptability.	NR.	**Impact on Research:** Co-created diabetes management plan tool for use in the clinic with teens and their parents.<br>**Impact on Young People:** NR.<br>**Impact on Policy:** NR.	**Conclusions:** Young people can be effectively engaged and involved in patient-centred research design. Important for patient-centred outcomes research to help people achieve personal goals and address diabetes distress.<br>**Lessons learned:** Young people with T1DM prioritize reducing family conflict and fitting into their social milieu over health outcomes.	Small number of PPI participants recruited from a single geographic area could affect the generalizability of findings. Individual responses could have been influenced by social desirability. Some participants unavailable for session 2 and only males attended this session.	NR.
Holmes et al. (2002), Australia.	**Aim:** To have opportunity for non-Aboriginal	**Term:** Participatory research, Peer interviewers.	**PPI (YP):** Young people recruited as peer reviewers	Defining research question and	NR.	**Impact on Research:** Use of peers helped to raise awareness of the study in the	**Conclusions:** Study contributed to a positive shift towards research among	NR.	This research model fits with the principles of self-determination

	researchers, Aboriginal health workers and young people to contribute together to the study design.	**Definition:** NR **Concepts/Theory:** NR.	[n=18; 12–25yrs, both sexes). **PPI (Others):** Adult community stakeholders.	developing questionnaire.		community, informed understanding of underlying issues and development of questionnaire. It was important that Kooris facilitate the discussions and interpret the results. **Impact on Young People:** Several Koori health workers and young people trained in research and public health skills. They gained confidence and now play important advocacy and management roles. **Impact on Policy:** NR.	Aboriginal policy makers and staff and increased the capacity. **Lessons learned:** Opportunities for genuine community involvement occur more readily when a study is based in a community organisation. Young Koori researchers played a key role in ongoing negotiation.		and offers one possible approach to improving Aboriginal health research practices.
Hunt et al. (2015), UK.	**Aim:** To use findings from the consultation to develop the research bid and develop collaborative relationships with the CYP, families and service providers.	**Term:** Service users, user involvement. **Definition:** User involvement includes consultation, collaboration and user-controlled research (Oliver et al., 2008). **Concepts/Theory:** NR.	**PPI (YP):** CYP (n=7; 13-18yrs, 6 male, 1 female; 5 wheelchair users, 6 had physical disabilities). **PPI (Others):** Parents, Service professionals.	Designing study and study logo.	NR.	**Impact on Research:** Consultations proved extremely helpful in shaping the research questions and research design. **Impact on Young People:** NR. **Impact on Policy:** NR.	**Conclusion:** Consultations proved extremely helpful in shaping the research questions and research design. **Lessons learned:** Important for researchers to gain an understanding of the formal and informal professional networks. Important to build close links with family users.	Difficult to determine when consultation efforts have been sufficient. Important to consult with those who receive services and those who might be excluded or currently not accessing.	
Kendal (2017), UK.	**Aim:** To ensure that support for youth should be informed by youth-led research.	**Term:** Participatory research. **Definition:** An approach, orientation, method, design or methodology aimed at co-production of knowledge between researchers and co-researchers (Bergold & Thomas, 2012). **Concepts/Theory:** NR	**PPI (YP):** Young co-researchers (n=11; 16-18yrs; n=10 in full-time education). **PPI (Others):** N/A.	Data collection and analysis.	NR.	**Impact on Research:** Young person influenced all stages of the study. **Impact on Young People:** NR. **Impact on Policy:** NR.	**Conclusions:** Participatory research underpinned all aspects of the project. Young people's analysis of their data and contribution to the writing up privileged their perspectives. **Lessons learned:** Better ratios of researchers to young people could have resulted in more one-to-one working and/or closer support. More time needed for some aspects of study to develop trust.	Number of participants was small and were mostly local, female and in full-time education.	Approach used may be a useful alternative to other participatory strategies which can be limited by low engagement. Need to explore strategies for establishing the quality and safety of self-care resources.
Larkins et al. (2013), UK.	**Aim:** To explore the impact that living in low income families has on disabled CYP's rights and to ensure that CYP led focus on which rights were focused on.	**Term:** Child rights-based approach (CRBA). **Definition:** CRBA starts from a commitment to achieving the rights and guiding principles of the relevant UN conventions; it informs children about their rights; it learns from children about infringements of their rights; it	**PPI (YP):** Disabled young people (n=43, 4-24yrs). Expert group (n=32, severe impairments). Steering group (n=11, ASD or visually impaired; 12-18yrs, 7 male, 4 female). **PPI (Others):** Professionals, families.	Involved in co-creation throughout the study.	Internal. No specific methodology stated. Evaluation of working with the steering and expert group. Researcher reflections.	**Impact on Research:** Young people decided on the themes explored and contributed substantially to the analysis of the findings. **Impact on Young People:** Enjoyment, fulfilment and development for CYP. Young people sent thank you cards' or reported that they really enjoyed the activities, they felt listened to, they helped each other, and they knew more about their rights. **Impact on Policy:**	**Conclusions: R**esearch illustrates a methodology for participatory research which has been co-created through dialogue and action by disabled CYP. It could be adapted to explore the impact of low income on the rights of other CYP. **Lessons learned:** NR.	The need to complete the research within four months, stay within budget and comply with University regulations.	NR.

		identifies barriers and mechanisms for giving greater effect to rights and it targets action to strengthen and monitor progress towards this. **Concepts/Theory:** Child rights-based approach.				Recommendations of report picked up by Office of Children's Commissioner with clearly stated recommendations for Government and local authorities.			
Liabo et al. (2018), UK.	**Aim:** To include the priorities and views of the people we research.	**Term:** Service user involvement. **Definition:** NR. **Concepts/Theory:** NR.	**PPI (YP):** Care leavers: (n=20, 16-24yrs, 12 female, 8 male). From UK (n=3). Unaccompanied asylum seekers from Central and East Africa (n=13), Eastern Europe (n=3) and Central Asia (n=1). **PPI (Others):** N/A.	All stages of review. Young people were trained in reviewing and were involved in every stage of the review, conference attendance and presentation.	NR.	**Impact on Research:** Young people influenced review topic, helped avoid stigmatising language, shifted focus to education and support interventions. Influenced researcher's knowledge, perspectives and ways of writing on looked after young people. Widened range of studies to be included. Influenced inclusion and exclusion criteria and search terms. Helped highlight hidden judgements and improved review transparency. **Impact on Young People:** NR. **Impact on Policy:** NR.	**Conclusions:** Young people's input challenged existing stereotyping and acted as a counterbalance to the researcher bias. Involvement in the screening process led to overachieving target. Involvement makes research more relevant and enhances transparency. If involvement is truly participatory, it will always influence the research. **Lessons learned:** Involvement is a give and take relationship based on ongoing deliberations, decision making and discussions. Involvement brings people together to discuss topics of importance: outcomes are reached through dialogue, joint learning and deliberation.	Potentially influenced by power relations. Systematic reviewing quite technical this may have presented barriers to young people involvement.	NR.
Lightfoot & Sloper (2003), UK.	**Aim:** To identify factors which can support involvement in ways which young patients find appropriate, and to prepare practical guidelines for NHS staff.	**Term:** Involvement work. **Definition:** Service user involvement in research, is the inclusion of experience-based experts in knowledge production, is key to developing useful, relevant and valid research (Collins & Evans, 2002). **Concepts/Theory:** NR.	**PPI (YP):** No age stated. **PPI (Others):** N/A.	Designing recruitment materials, data collection tools, dissemination	NR.	**Impact on Research:** NR. **Impact on Young People:** NR. **Impact on Policy:** NR.	**Conclusions:** Young people may have particular needs and value ways of working which are not readily accommodated within adult-centred strategies. **Lessons learned:** Involvement work has the potential to result in positive outcomes for the personal development of young patients and professional development of staff.	Characteristics of sample limit generalisability. Three groups of patients were not represented: younger children; those with severe impairments; and those from minority ethnic populations.	Further research with underrepresented groups may yield useful findings.
Locock et al. (2019), UK.	**Aim:** To understand to what extent service users might become involved in the development of trigger films and to consider how this might bring new insights to the process.	**Term:** PPI. **Definition:** Research is conducted with or by users, rather than to, for or about them. **Concepts/Theory:** Experience based co-design.	**PPI (YP):** Young people (n=6, 19–23yrs) with lived experience of depression or stroke. **PPI (Others):** Carers.	Analysing interview transcripts and developing film.	NR.	**Impact on Research:** Researchers discovered they were too attached to process, concerned with methods and that analysis means close immersion in large amounts of text. Users preferred conversational engagement in the analytic process. **Impact on Young People:**	**Conclusions:** The contribution to analysis can add a valuable layer to the process, ensuring the priorities of service users are firmly at the forefront. Reflections on the process led researchers to consider widening the definition of 'analysis' to include early conversation and guidance on	NR.	Commence involvement in analysis as early as possible in process.

						Some young people keen to get involved in further work. One wanted to take on extra analysis and decided to follow a career in research. **Impact on Policy:** NR.	the content of the analysis. **Lessons learned:** Involvement in analysis can be commenced at an earlier stage. Comparisons between what researchers and users see in the data are often more about important nuance and emphasis.		
Manning et al. (2018), England.	**Aim:** To identify and prioritise future research for CYP PICU survivors.	**Term:** Consultation and PPI. **Definition:** Not specifically defined but linked to INVOLVE (2012) guidelines. **Concepts/Theory:** NR.	**PPI (YP):** Young people n=24; 8 CYP aged 7–15 years (n=3 PICU survivors, n=2 PICU survivor siblings; n=3 other health experience). One young person (13yrs) helped to facilitate event. **PPI (Others):** Adult stakeholders.	Engagement in event.	NR.	**Impact on Research:** Identified priorities for future research and services. **Impact on Young People:** NR. **Impact on Policy:** NR.	**Conclusions:** Evidence of the value of meaningful PPI in the development of research priorities and health care services to ensure they are appropriate, relevant and acceptable. **Lessons learned:** It is important to include young people as some of their priorities differed to those of adults.	Sample was self-selected; therefore, transferability might be limited. Minority ethnic groups underrepresented. The homogeneity of the CYPs illness represents a limitation.	NR.
McLaughlin (2015), UK.	**Aim:** NR	**Term:** PPI. **Definition:** NR. **Concepts/Theory:** NR.	**PPI (YP):** Young people (n= approx. 14, 11–18yrs, roughly even split of female and male, experience of health conditions). **PPI (Others):** Parents, carers in FRAG.	Developing engagement materials.	NR for the Involving YP work. Independent for VIPER project using mixed methods evaluation indicated project was highly successful with co-researchers and co-producers being influential. Recruitment, training and support were effective, well planned and positively received. Young people's own life experiences helped to shape study and ensure a real resonance.	**Impact on Research:** Input of young people invaluable in shaping thinking; thoughtful, incisive comments helped us modify aspects of our approach. **Impact on Young People:** Feedback from YRA group suggests they found experience valuable "each of us loves being part of it… glad to give our own opinions for the better use and hope that they have influenced the way research is done". **Impact on Policy:** NR.	**Conclusions:** Clear evidence that involving CYP brings wide benefits; positive outcomes for young people, more tightly focused projects and outputs that are relevant to CYP, families and carers. **Lessons learned:** Need adequate time to undertake planning and preparation of research and participation activities. Need staff to support small group working. Important to plan sessions thoroughly and including a mixture of activities. Need time for informal discussion and for making adaptations.	Limited scope for the CYP to influence the research questions.	Allow more time when bidding for participatory projects so young people may act as co-researchers Train all personnel and participants in the different concepts and models. Allow sufficient time and funding for participation and go beyond the 'usual suspects'. Invite participation in all aspects of the work and listen to all views.
Mitchell et al. (2018), England.	**Aim:** To co-produce the recommendations	**Term:** PPI. **Definition:** NR. **Concepts/Theory:**	**PPI (YP):** Young people (12-22yrs) from various	Co-production activities planned for	NR.	**Impact on Research:** NR. **Impact on Young People:**	**Conclusions:** NR. **Lessons learned:** NR.	NR.	NR.

	for the model of care.	Patient experience framework.	existing advisory groups. **PPI Population (Other):** N/A.	whole study including protocol development, literature review, design of methods, materials, data collection tools, findings and dissemination.		NR. **Impact on Policy:** NR.			
Mitchell et al. (2019), UK.	**Aim**: To describe the approach to PPI with CYP for research in paediatric palliative care.	**Term:** PPI. **Definition:** PPI is the active involvement of patients and members of the public in the design and process of research. **Concepts/Theory:** NR.	**PPI (YP):** CYP (12-20yrs) from existing advisory groups. **PPI Population (Other):** N/A.	Designing research, dissemination, impact.	Internal. Confidential feedback from PPI group members about their experiences of being involved in this research gathered using 'Tell Me…' from 'RCPCH&Us Recipes for Engagement'.	**Impact on Research:** Changing the terminology. This influenced further research by team into language use in palliative care. **Impact on Young People:** Opportunities to engage in activities although no direct feedback from young people provided. **Impact on Policy:** NR.	**Conclusions:** The importance of incorporating PPI with CYP is well recognised. However, there is little guidance about how best to conduct such activity. **Lessons learned:** NR.	NR.	The framework that emerged is applicable in numerous contexts and could be used to identify and address ethical concerns pre-emptively, minimising the risk of harm to CYP while maximising the value of their contribution. More could be done to ensure equitable access to involvement opportunities as well as consistent or standardised training in the conduct of PPI for researchers.
Morton et al. (2017), England.	**Aim**: To provide a means of engaging multiple stakeholders in the prioritisation of school environment focused interventions to promote physical activity.	**Term:** Public involvement. **Definition:** Research carried out 'with' or 'by' members of the public rather than 'to', 'about' or 'for' them (INVOLVE). **Concepts/Theory:** NR.	**PPI (YP):** Young people (n=37, 12=17yrs). **PPI Population (Other):** Education and public health professionals.	Priority setting.	Internal. Participants (including adult PPI stakeholders) invited to provide informal free-text comments on the process of being involved in the prioritisation process.	**Impact on Research:** NR. **Impact on Young People:** NR. **Impact on Policy:** NR.	**Conclusions:** From the outset, the boundaries between 'public involvement' and 'research' were blurred. Although guidelines relating to public involvement and research ethics exist, we could not find information relating to the use of public involvement data in research publications. **Lessons learned: NR.**	NR.	Guidelines needed relating to the use of public involvement data in research publications.
Office of the Children's Commissioner (2014), England.	**Aim:** To help ensure that CYP's views and voices were at the centre of the research process.	**Term:** Young researchers. **Definition:** NR. **Concepts/Theory:** Participatory design.	**PPI (YP):** Young disabled people (n=4). **PPI Population (Other):** N/A.	Designing research, materials, data collection, analysis, report writing.	NR.	**Impact on Research:** Voices of CYPs were at the centre of the research process. Young researchers widened age range of target population, helped develop information sheets, ensured focus groups were accessible and engaging, identified games and activities to facilitate discussion. Supported	**Conclusions:** There are major obstacles and barriers which prevent disabled CYP realising their rights. **Lessons learned:** NR.	NR.	NR.

						analysis and dissemination.<br>**Impact on Young People:** NR.<br>**Impact on Policy:** NR.			
O'Hara et al. (2017), Ireland.	Aim: To form a PPI panel of young adults with T1D who would actively be involved in co-designing all aspects of the intervention.	**Term:** PPI.<br>**Definition:** Research carried out 'with' or 'by' members of the public rather than 'to', 'about' or 'for' them (INVOLVE).<br>**Concepts/Theory:** NR.	**PPI (YP):** Young people n=8. 18-25yrs, 3 male, 5 female).<br>**PPI Population (Other):** N/A.	Designing materials and data collection tools, dissemination of findings, co-applicant on grant.	Internal. No method stated. Feedback from CYP demonstrates they felt co-ownership of the design and development. Feedback from academic researchers highlights the invaluable role played by the YAP. Feedback from conference states young people's contributions as "greatest strength of the event".	**Impact on Research:** Funding awarded to study and YAP were first PPI panel to be recognised as official collaborators by the HRB.<br>**Impact on Young People:** Some YAP members asked the clinical research team to write letters of recommendation, help them with their CV and assist them entering academic competitions.<br>**Impact on Policy:** NR.	**Conclusions:** It is feasible and beneficial to include a PPI panel of young adults in health research. PPI research raises important issues related to the provision of patient care and involvement in research of those receiving care. Open dialogue and responsiveness to issues enabled the research team to recognise and address these issues. The study encountered common challenges in forming and progressing its PPI panel. The majority of barriers were successfully overcome.<br>**Lessons learned:** Forming the YAP required buy-in from both panel members and the rest of the research team. Expectations need to be managed. Avoid jargon.	The YAP consisted of only eight members and as such was not a representative group. Inadequate budget to pay members for their time.	NR.
Oliver et al. (2015), UK.	**Aim:** To elicit young people's reviews in relation to the review topics and draft findings in two configurative systematic reviews.	**Term:** Public involvement.<br>**Definition:** Outsiders influence research (Oliver et al, 2014).<br>**Concepts/Theory:** NR.	**PPI (YP):** Young people (n=approx. 24 across 2 groups, 12-17yrs).<br>**PPI Population (Other):** N/A.	Commenting on themes and final findings of the review.	NR.	**Impact on Research:** Young people identified important and/or missing themes from the synthesis resulting in studies and coding being revisited by researchers. Implications for research were developed by considering both priority areas for young people and explanations from the literature.<br>**Impact on Young People:** NR.<br>**Impact on Policy:** NR.	**Conclusions:** The review processes and products differed as a result of involving young people. Both systematic reviews became more relevant to young people.<br>**Lessons learned:** With more time for workshop preparation, a more considered approach could have been taken. There were advantages to having such an engaged, research and public health-literate group of young people to work with on the reviews.	Only one reviewer attended both workshops so young people's ideas filtered through this individual. Limited opportunity to question the young people about their ideas. Researchers did not have direct access to the raw data. Methods of involvement inevitably influenced the ideas elicited. Young people were older than the children in the studies, did not have a specialist interest which may have influenced their perceptions or limited their ability to empathise. May have been more	Involvement activities for systematic reviews need to be designed with the review stage, purpose and group being involved in mind.

								aware of health consequences and issues than most young people.	
Pavarini (2019), UK.	**Aim:** To develop a group where CYP are involved in shaping research.	**Term:** Co-production. **Definition**: Researchers, practitioners and the public work together, sharing power and responsibility from the start to the end of the project, including the generation of knowledge. (Hickey et al., 2018). **Concepts/Theory:** Co-production model.	**PPI (YP):** Young people (n=30, 15-18yrs) from wide range of backgrounds and schools. **PPI Population (Other):** N/A	Co-production at every stage of the research.	Internal. Facilitators periodically ask the young people to complete anonymous questionnaires and indicate what they consider to be priorities for the group moving forward. This feedback influenced structure/ activities of future sessions.	**Impact on Research:** Young people shifted research focus to one which they deemed more relevant to their daily lives. Developed peer-led interviews as a comfortable, engaging method giving young people greater sense of agency. Developed digital games to be used as engaging tools to collect data. Implemented more effective recruitment strategies, leveraging online platforms. **Impact on Young People:** Participation helped the young people gain both technical (research methods) and soft skills (confidence, teamwork). **Impact on Policy:** NR.	**Conclusions:** Adopting an open and reflective perspective can increase researchers' capacity to engage young people in ways that are meaningful, democratic and inclusive. **Lessons learned:** Important to have on-going evaluations on the impact of the group and reflections upon whether the group is mutually beneficial, and genuinely empowering for young people.	NR.	Researchers need to be reflective during the selection process and the running of the sessions, ensuring that different interests and voices are represented.
Perry & Carpenter (2016), UK.	**Aim:** To explore young people's views of appropriate outcome measures of the effectiveness of therapy and to develop a novel user-generated questionnaire.	**Term:** Co-researchers and co-participants. **Definition:** Research "with" rather than "on" young people (Reason and Bradbury Huang, 2005). **Concepts/Theory:** Action research or cooperative enquiry.	**PPI (YP):** Consultation group (10-18yrs). Pilot, young people (n=8, 10-17yrs, 4 male, 4 female, all white). **PPI Population (Other):** N/A.	Designing questionnaire.	NR.	**Impact on Research:** Young people developed and designed a new questionnaire (outcome measure) to capture experience/benefits of therapy; items included verbatim wording of their comments on therapy. **Impact on Young People:** NR. **Impact on Policy:** NR**.**	**Conclusions:** It is important to consider which voices are privileged and which voices are silenced. Healthcare providers consulting young people need to "listen and respond". **Lessons learned:** Importance of CYP being as involved as possible in the research process.	Attendance changed from workshop to workshop. There remained tensions and challenges in working collaboratively. Young people were generally very supportive and had positive experiences of counselling/ therapy and may be regarded as having less of a critical or objective voice. The adult coresearchers took a lead in some areas.	Outcome measurement needs to be as relevant and sensitive to CYP's experiences. Treating young people as co-researchers is recommended to anyone working in the field of CYP's mental health.
RCPCH (2012), UK.	**Aim**: To undertake consultations with parents and carers and CYP themselves, to derive the issues important to this group and to design and test a survey with the target user group.	**Term:** Patient participation. **Definition:** NR. **Concepts/Theory:** NR.	**PPI (YP):** CYP in focus groups (5-18yrs). **PPI Population (Other):** Parents and carers of 0-8yr olds	Developing survey designed 'by the child for the child'.	NR.	**Impact on Research:** Young people identified and ranked the important domains of healthcare which influenced weighting of the survey questions, topics and domains. Identified key domains they were concerned. Cognitive testing highlighted the need for separate versions of the	**Conclusions:** This collaborative project was able to develop a robust tool to measure the experience of paediatric patients; this incorporated children's views. **Lessons learned:** NR.	NR.	NR.

						survey.<br>**Impact on Young People:** NR.<br>**Impact on Policy:** NR.			
Sheridan et al. (2019), UK.	**Aim**: To seek input, inform decision-making and review study documentation and tools.	**Term:** PPI.<br>**Definition:** Research … carried out 'with' or 'by' members of the public rather than 'to', 'about' or 'for' them (INVOLVE).<br>**Concepts/Theory:** Responsive & managerial public involvement approach.	**PPI (YP):** Young people (n=3, 19-24 yrs, 2 female, 1 male) with long-term health conditions. Plus members of the Liverpool GenerationR Young People's Advisory Group.<br>**PPI Population (Other):** Mothers of young people with long term health conditions.	Informing decision making throughout study and review materials.	Internal. No methodology stated although all feedback was recorded in full and summarised. Data regarding the impact of the YPAG, and the strengths and difficulties of involvement were generated. No formal data analysis was undertaken.	**Impact on Research:** Young people improved the clarity of study documentation and challenged the view of the researchers. However, when materials were already approved by Research Ethics Committees, young people's suggestions noted but not incorporated**.**<br>**Impact on Young People: NR.**<br>**Impact on Policy:** NR.	**Conclusions:** The research team felt the involvement of the YPAG was a positive addition. The young people felt the study team appreciated the importance of PPI which enabled them to express opinions openly and honestly.<br>**Lessons learned:** The importance of treating the young people with the same courtesies as other members to ensure they felt valued. Careful and early planning can help avoid logistical challenges.	NR.	Honest and open communication, varied opportunities and a recognition by the research team of the importance of PPI is essential in projects.
Snodin et al. (2017), England.	**Aim:** To ensure that the language and content of the CYP's study documents were appropriate and accessible and would promote recruitment and assent/consent.	**Term:** PPI, Consultation.<br>**Definition:** NR.<br>**Concepts/Theory:** NR.	**PPI (YP):** Young people (n=7, 5-12yrs) with wide range of health experiences.<br>**PPI Population (Other):** Parents, teacher.	Research design.	NR.	**Impact on Research:** Young people helped refine focus and objectives of study, rejected some proposed methods of data collection but supported others, ensured study documentation relevant and accessible and influenced terminology used to describe clinical holding that would be accessible for children.<br>**Impact on Young People:** NR.<br>**Impact on Policy:** NR.	**Conclusions:** Conversation rather than data is at the heart of user involvement in analysis.<br>**Lessons learned:** Elicit user reflections at the start of analysis and use this as a guide to direct the researcher's gaze.	NR.	Researchers should engage in robust consultation as the process undertaken for this study offered an opportunity to explore intended research approaches with CYP with similar experiences to the intended study participant.
Taylor et al., 2015 UK	**Aim**: To involve young people in naming and branding the study which may then contribute to successful participation and retention.	**Term:** PPI.<br>**Definition:** active inclusion of patients, carers, service users, and stakeholders and may be defined as research being carried out 'with' or 'by' members of the public rather than 'to', 'about' or 'for' them.<br>**Concepts/Theory:** NR.	**PPI (YP):** Young people (n=9, 17-26yrs) with cancer. Plus (n= approx. 200) at teenage cancer conference.<br>**PPI Population (Other):** N/A	Naming and branding of study.	Internal. No methodology although evaluation took place after each of the six of the workshops.	**Impact on Research:** Young people changed name and branding of study; although impact not known it is hypothesised this may have positively influenced acceptability of the study resulting in higher acceptance rates. Anecdotally, young people and healthcare professionals responded favourably to change.<br>**Impact on Young People:** NR.<br>**Impact on Policy:** NR.	**Conclusions:** Acceptance and retention to study is higher than anticipated (80% versus 60%), this may be related to the integral PPI strategy.<br>**Lessons learned:** Involving young people with developing brand and logo should be carried out prior to engaging with healthcare professionals to prevent confusion around study identity.	Branding process commenced midpoint through gaining regulatory status.	Start branding process early.
Tume et al. (2016), England.	**Aim**: To determine the acceptability, relevance and importance of the trial to parents and children and	**Term:** Consultation/ PPI.<br>**Definition:** NR.<br>**Concepts/Theory:** NR.	**PPI (YP):** Young people including (n=1, 15yrs, male) with PICU experience and (n=13, 9-18yrs,	Designing study and materials.	NR.	**Impact on Research:** Pragmatic, useful, insights gained to inform the trial design.<br>**Impact on Young People:**	**Conclusions:** Trial objectives were deemed important and relevant. Parents and young people did not consider written informed consent to be necessary rather awareness of	Small number of parents and young people due to recruitment problems.	Novel recruitment approaches required for work in this field.

	determine parent/patient-focused outcome measures and ascertain their views on informed consent in a cluster RCT.		average 15yrs, 10 females, 3 males) with no PICU experience.<br>**PPI Population (Other):** Parents (n=2).			NR.<br>**Impact on Policy:** NR.	unit participation in the trial was important with the opportunity of opting out.<br>**Lessons learned:** Novel recruitment measures need to be considered for future work in this field; recruitment of ex-PICU population challenging.		
Walsh et al. (2018), Ireland.	**Aim:** To increase the likelihood of developing a feasible, implementable, applicable and effective intervention	**Term:** PPI, Stakeholder,<br>**Definition**: PPI occurs “when individuals meaningfully and actively collaborate in the governance, priority setting, and conduct of research, as well as in summarising, distributing, sharing, and applying its resulting knowledge” (de Wit et la., 2013).<br>**Concepts/Theory:** user-centred design development	**PPI (YP):** Young people (n=8, 18-25yrs).<br>**PPI (Others):** N/A.	Contributing to systematic review, development of materials and data collection tools, organising committee for symposium, co-applicant on grant.	NR.	I**mpact on Research:** NR.<br>**Impact on Young People:** NR.<br>I**mpact on Policy:** NR	**Conclusions:** PPI approach was instrumental in moving from theory to operationalising core intervention components.<br>**Lessons learned:** Importance of managing expectations of the YAP and research team in terms of what is feasible, implementable, and how quickly change can happen. Importance of expectation management being undertaken by a neutral ‘knowledge broker’	NR.	Engaging with key stakeholders is recommended as best practice for effective intervention development.
