# Supplementary material for: Children and young people’s contributions to public involvement and engagement activities in health-related research: A scoping review
Source: PLoS One. 2021 Jun 9;16(6):e0252774. doi: 10.1371/journal.pone.0252774 (PMC8189547; doi:10.1371/journal.pone.0252774)
Supplement: Attachment — Submitted filename: Response to Reviewers.docx [file pone.0252774.s003.docx]

**PLOS One**

**Article: Children and young people’s contributions to public involvement and engagement activities in health related research: a scoping review.**

Dear Catherine J Evans,

First, we would like to thank all the reviewers for their careful reading of the manuscript and their constructive remarks. We were impressed by the thoroughness of the reviews and appreciate the time and thoughtfulness that all four reviewers have put into the feedback. We have provided a detailed list of our responses to the reviewers’ comments below and note the changes we have made to the manuscript.

We hope the reviewers agree that by addressing their comments we have produced a much stronger paper.

Many thanks again for considering our paper for publication.

Best wishes,

Alison Rouncefield-Swales (corresponding author; writing on behalf of all authors).

**Line references in the responses refer to the Line Numbers in the CLEAN/untracked version.**

| Reviewer #1: Thanks for the opportunity to review this article, which is a well written scoping review of children and young people's PPIE activities in health related research. It addresses an important issue and adds to the growing literature in this area. There are a few suggestions that would enhance the article: | Thank you for your positive and well-considered review. |
| --- | --- |
| 1. The number of authors/reviewers is confusing. There are 6 listed, but the text refers to five? It is also incorrect to state that the authors contributed equally to the work, when only two authors undertook the initial screening process. | 1. There were five reviewers of articles for this scoping review with any two of the five undertaking the initial screening and the extractions were split between all five reviewers. The Family Engagement officer played a key role in helping define the review and in bringing together the discussion. We have recognised this as an equal contribution. |
| 2. In the introduction, a reference to CYP's tokenistic involvement in research and Hart's ladder of participation would be helpful, particularly as this is appropriately critiqued later on. | 2. We have included “with their involvement being tokenistic, controlled and their decision-making limited as can be seen in studies that only operate at the bottom of Hart’s ladder of Participation” at the end of Line 87-88. |
| 3. The aims of the review should be at the end of the introduction rather than at the beginning of the methods. | 3. We have moved the aim and objectives to the end of the introduction, Lines 111 – 114. |
| 4. In the study selection, what is the justification for limiting the search to papers published after 1 January 2000? | 4. We wanted the findings of the review to provide a contemporary overview (broadly the last 20 years) of the involvement of CYP in PPIE activity. Although PPI in research started the 1970s, our selection of 2000 as a start date aimed to provide a detailed, in-depth contemporary consideration of the field which has broadly shifted to more inclusive practices with children and young people. |
| 5. Inclusion criteria - does including systematic reviews present the possibility of double reporting? Would it not be better to search the included studies of relevant systematic reviews? | 5. We included systematic reviews in order to search the included studies of relevant systematic reviews. We have changed the wording in Table 1. |
| 6. Exclusion criteria - I don't understand what is meant by 'Articles in press'. | 7. Articles in Press are articles which have been accepted for publication but have not yet been published. This was only relevant for one database – Scopus. |
| 6. On page 8, the phrase 'a good fit' feels unscientific - did papers meet the inclusion criteria or not? | 8. We have changed the wording on Line 184. |
| 7. The development of the QRIPPAT tool is to be applauded. However Table 4 would benefit from the papers being referenced and a score of the quality of each paper would enable comparison. | 9. We have referenced the papers in Table 4 and updated to include a score. We recognise that assigning scores to quality appraisal can be seen as contentious, especially if the scoring system is unweighted or unvalidated yet we understand the value of scores as part of the critique of a paper. We have equally weighted each element of the QRIPPAT and assigned a score of 1, giving a range of 0-10, where 0 is lowest score for quality and 10 the highest quality score). See Lines 209 – 215. |
| 8. The paper would benefit from a proof read, there are some typos.  On the whole, it is a great paper and like all good papers, has forced me to reconsider my own practice in this area. Well done. | 9. We have proofread the whole article and addressed typos. |

| Reviewer #2: This is a timely and exhaustively prepared piece of work with a solid method. It follows all the right current trends (e.g. GRIPP2), it is well-read (e.g the use of Levac was excellent) and is a well-intentioned piece of work. This is important and gives INVOLVE much to think about whilst reviewing its scope. It also enables researchers to see with clear evidence what works and works well for PPIE. Good article which needs disseminating widely. | Thank you for your positive review. |
| --- | --- |

| Reviewer #3: Many thanks for the opportunity to review this article. | Thank you for your comprehensive and constructive review. |
| --- | --- |
| 1. It provided interesting results however I had some concerns over the distinction between Patient and Public Involvement (PPI) and Public Engagement (PE). The difference between "involvement" and "engagement" is briefly highlighted but I would disagree that it’s now a broad term used by many health organisations. The use of these terms is a continuing debate in this area and many consider people to use the terms incorrectly often referring to public engagement as PPI or labelling PPI as public engagement, when they are very different activities. It would be better for the authors’ say they are choosing to use the term PPIE throughout the article to encompass both PPI and PE activities. | 1. We accept your helpful reflections and have updated the introduction Lines 63-66 to better reflect our usage the of term PPIE throughout the article to encompass both PPI and PPE activities. |
| 2. In addition, it felt like some of the current debates in this area weren’t fully explored or acknowledged: how impact should be measured, payment for PPI/PE. There was also no mention of the NIHR UK Standards for Public Involvement in Research and how any of these studies met (or didn’t meet) these. | 2. We acknowledge that we were unable, due to word limits, to explore fully or acknowledge all areas of PPIE which are of interest and are frequently debated. We have updated the introduction to include reference to the NIHR UK Standards for Public Involvement in Research (Lines 66 – 68). We recognise that the UK Standards could have been used as a framework for understanding what good public involvement, however we feel that the GRIPP offered a greater synergy with the aims of this review. |
| 3. The articles would benefit from being carefully checked for spelling grammar mistakes as there were a few. | 3. We have proofread the whole article and addressed typos. |
| Introduction: |  |
| 4. Line 61: PPI is widely accepted as referring to “Patient and Public Involvement” rather than “Public and Patient Involvement” | 4. Amended. |
| 5. Line 61: “The term PPI rather than for public and patient involvement…” is there a word missing as this sentence isn’t clear. | 5. Amended. |
| 6. Line 66: while there is evidence that meaningful PPIE is an essential part of research, there is (unfortunately) still a reluctance by many to include it in their research | 6. Amended. |
| 7. Line 69: unfortunately many researchers/clinicians do see PPIE as an optional extra. It is not compulsory on all funders’ application forms. Even with those that do include PPIE requirements, there is a gap between what is include on application forms and what happens in practice. | 7. Amended. |
| Methods: |  |
| 8. It would be interesting to include how many of the ways for measuring the impact of PPIE view PPIE as something that can be evaluated, for example and intervention | 8. Thank you, this is an interesting point. While not an explicit question we asked within the review we make passing reference to this in the discussion. The majority of studies only reported the impacts of PPIE anecdotally so we could not comment on this in the review. We recommend improvements to the reporting of PPIE evaluations in the discussion as a result of this gap. |
| 9. Line 99: “PPI” is used here rather than “PPIE”? | 9. Amended. |
| 10. Line 111: The definition given is the most widely known definition of PPI in the UK, but it is not PPIE. Engagement is not “research carried out with or by children and young people”. Engagement, as defined in the introduction, is “where information and knowledge about research is provided and disseminated”. | 10. We have amended Lines 125-127. |
| 11. Line 123: why was 25 chosen as the age limit for “young people” | 11. Please see our reasoning for the age range outlined in Section 3: study selection – Lines 155 – 158. |
| 12. Line 123: here public involvement is defined correctly but public engagement is missed out? | 12. We have corrected this omission. |
| 13. Line 126: authors’ initials missing “XX” | 13. Amended. |
| 14. Line 139: why was 1 January 2000 chosen as the earliest date when searching? | 14. We wanted the review to provide a contemporary overview of the involvement of CYP in PPIE activity. PPIE in research started the 1970s, but became more of a priority since the early 2000s |
| 15. Line 147, 148, 149, 167, 183: authors’ initials missing “XX” | 15. Amended. |
| 16. Line 206: How did the scope change after involvement from the service user? | 16. We have clarified the impact of the discussions in Line 231 - 234. |
| 17. Line 209: Family Engagement Officer’s initials missing | 17. Amended. |
| 18. Line 209: It would be interesting to hear more about changes made after challenges from the Family Engagement Officer. | 18. Amended. |
| 19. Line 213: “Servicer user” is without a hyphen in line 205 but with a hyphen in line 213. It would be better if these could be the same for consistency | 19. Changed for consistency. |
| Findings: |  |
| 20. Line 219: the “are” is not included in the objectives in line 100 | 20. Amended. |
| 21. Line 324: Line 404 – “Were” is in capitals and doesn’t need to be | 21. Amended. |
| 22. Line 408: Good to include the negative impact as well as positives. Did the study report how they support the CYP through this? | 22. The study reported that they wrote a letter to the store owner (the study was about tobacco sales) but did not send this after seeking legal council. The incident led to improvements to safety protocols for the co-researchers. These included the co-researchers carrying a handout about the study and being prepared to explain the study using an ‘elevator pitch’, and restricting restricting data collection to areas where they were known. We have added the phrase “resulting in changes to the study safety planning” at the end of Line 436. |
| 23. Line 411: the word “people” is missing from the title | 23. Amended. |
| 24. Line 473: Should this be PPI or PPIE here? | 24. Amended. |
| Discussion: |  |
| 25. It would be interesting to see more information included on why exactly there is difficulty in evaluating the impact of PPIE in research | 25. There is currently little evidence regarding the difficulties in evaluating the impact of PPIE in research and we hope this paper makes a contribution, but further research is required. |
| 26. It’s unusual to have some of the headings in the discussion section posed as questions and that the headings are the same as the ones used in the findings section. | 26. Thank you for your comment, we have changed the findings headings. |

| Reviewer #4: The authors present a well-conducted scoping review on patient and public involvement and engagement (PPIE) with children and young people in health-related research. Their findings highlight some important areas for improved practice around reporting characteristics of PPIE members, involving people throughout the research cycle, and more rigorously capturing impact. Below are some suggestions intended to strengthen the manuscript. | Thank you for your constructive review. |
| --- | --- |
| Abstract |  |
| 1. There is currently some blurring between the methods/findings (e.g. number of studies identified is in the methods) and findings/conclusions (e.g. there are authors reflections under findings). | 1. We have edited and refined the methods, findings and conclusions sections. |
| 2. Please consider being more specific in the findings and conclusions. For example, it’s currently it’s not clear what was variable about their involvement, and the conclusions feel quite general (rather than linked to the specifics of your interesting review findings). | 2. Please see above regarding edits and refinement. |
| 3. It might be more meaningful to present the number of records after duplicates were removed (n=9805) to reflect the number screened | 3. We have updated the numbers as suggested. |
| Introduction | |
| 4. I’m not sure I’d agree that PPIE is now used instead of PPI – it depends on if people are conducting ‘engagement’ as well as ‘involvement’ activities. The NIHR defines these as quite different activities: https://www.invo.org.uk/public-involvementparticipationengagement-in-research/. It’s fine if this review includes both engagement and involvement, but the definition in the methods only covers involvement. Please could this be clarified? | 4. We have clarified our use of the terms PPI and PPIE in the introduction. |
| 5. I was puzzled by the focus on reporting in the introduction as this does not relate to the review objectives – please consider whether some of this may be better suited to the discussion. | 5. This is a really helpful point. Our interest in reporting is due to this providing the evidence for the review. While we accept this does not clearly relate to the objectives, we feel it is worth giving prominence in the introduction due to the variability of reporting found. Furthermore, reporting quality was an important methodological concern for the review. |
| Methods | |
| 6. Please could the authors clarify the types of grey literature which were used? Searching for grey literature is described, but then the exclusion criteria excludes unpublished theses and articles in press. | 6. While we excluded unpublished theses and articles in press, our search of Grey literature led to the identification of a range of research reports including the studies by McLaughlin 2015; Office of Children’s Commissioner 2014; and RCPCH 2012. |
| 7. Please consider holding back on findings (e.g. the number of papers screened / included, the reporting quality assessment) until the results section. | 7. We have removed these references in the methods section. |
| 8. Please can the authors clarify why the GRIPP-2-LF was not sufficient to assess reporting quality? | 8. We felt that as the GRIPP2-LF was designed as guidance for reporting it was not directly applicable to assess the quality of reporting. Furthermore, while 25 papers purely reported on PPIE with the remaining 15 papers reporting PPIE in a small section of their paper, this meant that GRIPP2-LF was not a suitable tool as it would result in an unfair bias towards the papers solely focusing on PPIE. |
| 9. Page 12 line 192 – Please consider rephasing as this currently feels as though it conflates reporting quality with the quality of the work. | 9. We edited the sentence (Lines 213 – 215) to reflect the tool reports on quality of reporting not quality of study. |
| Results | |
| 10. The results section currently includes a lot of information, and it’s not always clear that these relate to the review objectives – e.g. the sections on PPIE terminology, definitions and theoretical influences; methods to evaluate PPI, limitations of involvement. Please consider whether these are necessary to include, as they may distract from the core review findings. | 10. Thank you for the suggestions. We have removed some sentences which we feel are repetitive. While some of the results sections do not relate directly to the objectives of the review, they are important in terms of providing the context and relate to the reporting measures listed in GRIPP-2. Furthermore, the wider variation across the studies provides an unintended but important finding of the review. |
| 11. Table 4: This information is well-summarised in the text, I wonder if the table itself would be better as a supplement (and potentially swapping this for the PRISMA flow chart, which would be helpful in main text). | 11. We feel it is beneficial for the reader to have Table 4 in the main article. We understand that the PRISMA flow chart will be available in the main text. |
| 12. Page 20 lines 297 to 304 lists how many were involved in each study. As the results section is already quite long, this might not be necessary to include in the main text. | 12. Thank you. We feel this helps provide context but we have shortened this section. |
| 13. Page 20 lines 311 notes that 28 studies recruited people with specific experience. As the objective for this section is ‘how’ people are recruited, please could more detail be provided? Readers may find this help to provide ideas of how they can reach out to PPIE members themselves. | 13. We have included further information on recruitment. See lines 338 – 344. |
| 14. There are a few things in the results that may be better placed in the methods. For example, page 20 lines 320-324 on how the NIHR research process model was used to categorise findings, and page 24 line 394 stating that impacts were taken directly from authors reporting and not inferred. | 14. We have moved the lines suggested into Method//Stage 5. See Lines 223 – 227. |
| Discussion |  |
| 15. The first couple of sentences of the introduction might not be needed as they repeat aspects of the introduction/aim. It was also unclear how bringing in the comments on the hierarchical approach related to the findings. | 15. We have made earlier reference to hierarchical approaches and so feel it is relevant to retain these sentences here. |
| 16. I worry that although the point about reporting is important, this was not part of the review objectives, and the amount of focus on this aspect is drawing away from the other important findings (e.g. about gaps in involvement across the research cycle, about the need for more rigorous evaluation activities). | 16. This is a very useful point. While reporting of PPIE was not an overall objective, it is important to include in the discussion as the variability in reporting of PPIE impacted on what conclusions could be drawn from the review. |
| 17. I’d be hesitant to highlight that only two studies reported impact on policy, as the search strategy for the review was particularly focused on PPIE in the context of health research (rather than searching for reports of involvement in health research and policy, for example). | 17. This is an excellent point. We have now made clear reference to our search strategy being focused on health research. Line 639-640. |
| 18. As the authors have done well in the earlier discussion paragraphs so make the implications quite clear, the 'implications' paragraph feels like it contains some repetition. | 18. We have made some edits to reduce repetition. |
| 19. I would suggest recommending further work to develop the QRIPPAT for potential use to assess reporting quality, rather than recommending use in it’s current form. It’s important to note that the EQUATOR group recommend reporting guidelines are developed using a formal process, including a consensus exercise: <https://journals.plos.org/plosmedicine/article?id=10.1371/journal.pmed.1000217#s1> | 19. Thank you. This is a suggestion we will pursue to develop the potential of the tool further. |
| 20. Similar to the abstract, please revise the conclusions to more specifically summarise the review findings. In line with above, I’d remove the recommendation of using the QRIPPAT as this was not the focus of this project, and it was not developed with an explicit methodology. | 20. We have revised the conclusions. |
